# Changing forest water yields in response to climate warming: results from long-term experimental watershed sites across North America

**DOI:** 10.1111/gcb.12615

**Published:** 2014-06-14

**Authors:** Irena F Creed, Adam T Spargo, Julia A Jones, Jim M Buttle, Mary B Adams, Fred D Beall, Eric G Booth, John L Campbell, Dave Clow, Kelly Elder, Mark B Green, Nancy B Grimm, Chelcy Miniat, Patricia Ramlal, Amartya Saha, Stephen Sebestyen, Dave Spittlehouse, Shannon Sterling, Mark W Williams, Rita Winkler, Huaxia Yao

**Affiliations:** 1 Department of Biology, Western University1151 Richmond St., London, ON, N6A 5B7, Canada; 2 Department of Geography, College of Earth, Ocean, and Atmospheric Sciences, Oregon State UniversityCorvallis, OR, 97331, USA; 3 Department of Geography, Trent University1600 West Bank Drive, Peterborough, ON, K9J 7B8, Canada; 4 USDA Forest Service, NRSP.O. Box 404, Parsons, WV, 26287, USA; 5 Natural Resources Canada, Canadian Forest Service, Great Lakes Forestry Centre1219 Queen St. East, Sault Ste. Marie, ON, P6A 2E5, Canada; 6 Department of Civil and Environmental Engineering, University of Wisconsin-Madison1552 University Ave., Madison, WI, 53726, USA; 7 USDA Forest Service271 Mast Rd., Durham, NH, 03824, USA; 8 Colorado Water Science Center, US Geological Survey, MS 415 Denver Federal CenterDenver, CO, 80225, USA; 9 Rocky Mountain Research Station, USDA Forest Service240 West Prospect Rd., Fort Collins, CO, 80526, USA; 10 Center for the Environment, Plymouth State UniversityPlymouth, NH, 03264, USA; 11 School of Life Sciences, Arizona State UniversityTempe, AZ, 85287, USA; 12 Coweeta Hydrologic Laboratory, Southern Research Station, USDA Forest ServiceOtto, NC, 28763, USA; 13 Fisheries and Oceans Canada, Freshwater Institute501 University Crescent, Winnipeg, MB, R3T 2N6, Canada; 14 Global Water for Sustainability Program, Florida International UniversityMiami, FL, 33199, USA; 15 Center for Research on Ecosystem Change, USDA Forest Service1831 Hwy 159 East, Grand Rapids, MN, 55744, USA; 16 Lands and Natural Resource Operations, BC Ministry of Forests1520 Blanshard St., Victoria, BC, V8W 9C2, Canada; 17 Department of Earth Science and Environmental Science, Dalhousie University1355 Oxford Street, Halifax, NS, B3H 4R2, Canada; 18 Department of Geography, University of Colorado-BoulderBoulder, CO, 80309, USA; 19 Lands and Natural Resource Operations, BC Ministry of Forests441 Columbia Street, Kamloops, BC, V2C 2T3, Canada; 20 Dorset Environmental Science Centre, Ontario Ministry of the Environment1026 Bellwood Acres Rd., Dorset, ON, P0A 1E0, Canada

**Keywords:** Budyko curve, catchments, climate change, elasticity, evapotranspiration, forest, precipitation, resilience, water yield

## Abstract

Climate warming is projected to affect forest water yields but the effects are expected to vary. We investigated how forest type and age affect water yield resilience to climate warming. To answer this question, we examined the variability in historical water yields at long-term experimental catchments across Canada and the United States over 5-year cool and warm periods. Using the theoretical framework of the Budyko curve, we calculated the effects of climate warming on the annual partitioning of precipitation (*P*) into evapotranspiration (ET) and water yield. Deviation (*d*) was defined as a catchment's change in actual ET divided by *P* [AET/*P*; evaporative index (EI)] coincident with a shift from a cool to a warm period – a positive *d* indicates an upward shift in EI and smaller than expected water yields, and a negative *d* indicates a downward shift in EI and larger than expected water yields. Elasticity was defined as the ratio of interannual variation in potential ET divided by *P* (PET/*P*; dryness index) to interannual variation in the EI – high elasticity indicates low *d* despite large range in drying index (i.e., resilient water yields), low elasticity indicates high *d* despite small range in drying index (i.e., nonresilient water yields). Although the data needed to fully evaluate ecosystems based on these metrics are limited, we were able to identify some characteristics of response among forest types. Alpine sites showed the greatest sensitivity to climate warming with any warming leading to increased water yields. Conifer forests included catchments with lowest elasticity and stable to larger water yields. Deciduous forests included catchments with intermediate elasticity and stable to smaller water yields. Mixed coniferous/deciduous forests included catchments with highest elasticity and stable water yields. Forest type appeared to influence the resilience of catchment water yields to climate warming, with conifer and deciduous catchments more susceptible to climate warming than the more diverse mixed forest catchments.

## Introduction

Since the Industrial Revolution, warmer air temperatures have been observed at continental scales (Jansen *et al.*, 2007). The effects of climate warming on water yield from headwaters are of great concern given their key role as water supply source areas (National Research Council, 2008). Long-term meteorological and hydrological records in headwater catchments, initiated to investigate management effects on hydrological fluxes in the early 20th century, are increasingly valuable for exploration of the effects of climate warming on water supplies. These data indicate that water yield response to climate warming varies among biomes (Jones *et al.*, 2012). This variability highlights the difficulties of predicting water yield response to climate change and its consequences for downstream water supplies (Bates *et al.*, 2008).

Different responses among catchment water yields to climate warming may reflect differences in resilience. Resilience concepts in environmental studies were first introduced by Holling (1973), who defined a resilient ecosystem as one that is able to absorb change while maintaining ecosystem function. Holling (1996) went on to distinguish between the concepts of engineering vs. ecological resilience. Engineering resilience suggests that a system may exist in only one stable equilibrium state; to measure such a system's resilience, one must determine its resistance to change and the time needed to return to the equilibrium state. Ecological resilience suggests that a system may exist in multiple stable equilibrium states; resilience in this case is measured as the magnitude of change an ecosystem can absorb before it shifts from one stable state to another stable state. While humans may deem some equilibrium states more desirable or valuable than others, the assumption is that each stable state is ecologically functional. Therefore, the main difference is that engineering resilience implies a single state (the system may be displaced from that state but if it is resilient, it will return to it), whereas ecological resilience implies a system flip among two or more stable states, all of which reside in a landscape of possible alternatives, and different disciplines have adopted different definitions to describe resilience (Brand & Jax, 2007).

Catchment scientists have recently started to apply resilience concepts to hydrological sciences. In this article, we adopt the concept of hydrological resilience (Gerten *et al.*, 2005): the ability of a catchment to absorb change and maintain or quickly regain hydrological function. This definition effectively refers to engineering resilience, which is more appropriate than ecological resilience for exploring the impact of climate warming on catchment water yields. Hydroloigcally resilient catchments are those with stable (operating within a range of natural variability, Poff *et al.*, 1997) and/or predictable water yields in face of changing environmental conditions. Catchments that lack hydrological resilience can be problematic. Human communities have often developed on the basis of historical water yields, and for this reason, substantial changes to water yields place these communities at risk.

**Fig 1:**
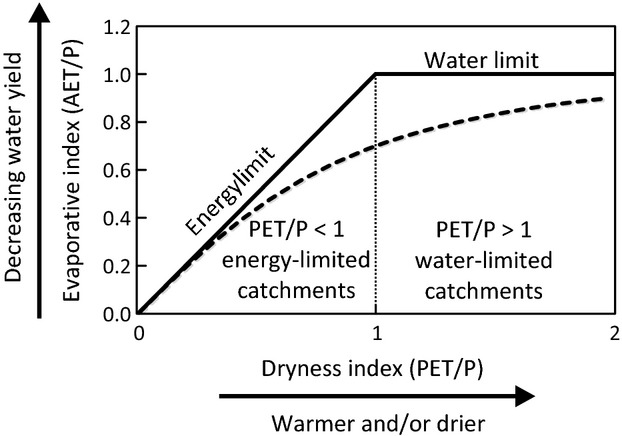
A Budyko diagram (evaporative vs. dryness index). The solid lines represent energy and water limits to the evaporative index, and the dashed line represents the original theoretical Budyko curve (after Budyko, 1974).

Recent catchment hydrological studies have used a Budyko curve (Fig.1, Budyko, 1974) approach to examine the interactions of climate, vegetation and water yield (e.g., Wang & Hejazi, 2011; Gentine *et al.*, 2012; Williams *et al.*, 2012; Troch *et al.*, 2013), but none of these studies uses long-term data from forested headwater catchments to explore the hydrological resilience of water yields to changing climate. We use the Budyko curve to explore the concept of hydrological resilience. This well-known curve describes the relationship between a catchment's potential evapotranspiration (PET) and its actual evapotranspiration (AET), each normalized by precipitation (*P*) – i.e., the curve describes AET/*P* (evaporative index, EI) as a function of PET/*P* (dryness index, DI). Budyko defined two catchment states, with evapotranspiration (ET) being limited by either energy supply or water supply. Climate determines the drying power of the atmosphere (net radiation and vapor pressure deficit) and the supply of water in the catchment (intercepted by the canopy or stored on ground surface or in soil) both of which influence ET. A value of DI < 1 indicates a humid, energy-limited catchment, whereas a value of DI > 1 indicates a dry, water-limited catchment. A catchment can be plotted on the Budyko curve based on its DI and EI. Paired DI and EI values based on long-term monitoring data from North American forested headwater catchments place the catchments on or near the Budyko curve (Jones *et al.*, 2012). Long-term offsets from the curve are likely due to unaccounted-for site characteristics such as vegetation type (Zhang *et al.*, 2001), soil type (Wang *et al.*, 2009), water storage capacity (Milly, 1994), or timing of water recharge (Potter *et al.*, 2005). We conceive of forested headwater catchments as exhibiting hydrological resilience because they hover around an attractor state defined by the Budyko curve but occasionally deviate due to a climatic variability or climatic extremes. Ultimately, though, they return to that attractor.

An underlying assumption of the Budyko approach is that over the long-term, mean annual *P* can be predictably partitioned into ET and water yield (*Q*): *P* = ET + *Q*. The larger the DI (Fig.1), the greater the proportion of precipitation that is partitioned to ET and the less that is available for discharge (water yield). A catchment that plots on the left-hand side of the curve will have greater water yield (smaller EI) than those catchments that plot on the right-hand side of the curve (larger EI). However, the Budyko curve may also provide a useful framework for developing a predictive understanding of how catchments respond to changing climatic conditions. For an individual catchment, we ask the questions: As DI (climate) changes, how does EI (water partitioning) respond? And do the DI and EI points move along the Budyko curve or do they deviate from the curve? A catchment that plots above (below) the curve is allocating more (less) water than predicted to ET and is yielding less (more) than predicted in the form of runoff. Relative to the Budyko curve, we define hydrological resilience as the ability of a catchment to absorb the effects of climate change and still maintain hydrological function as predicted by the curve. We suggest that hydrologically resilient catchments need not be fixed at a specific location on the Budyko diagram but that they do need to adapt to changing conditions such that their DI and EI points keep them near the Budyko curve.

To the extent that recent climate warming has manifested as increased atmospheric drying power (increased DI), we would expect that hydrologically resilient energy-limited catchments may be changing their allocations of *P* such that the proportion going into ET is increasing (increased EI) at the expense of water yield. A number of mechanisms operating over a range of scales could be involved, including (a) stomata closing in response to the increase in drying power; (b) forests accessing water stored in riparian areas, wetlands and lakes; or (c) forests reallocating water between evaporation (from intercepted or stored water) and transpiration, with some tree species reallocating more toward one than the other. All of these, as well as other factors like changes in timing and magnitude of precipitation (including partitioning of rain vs. snow) and changes in vegetation and soil composition, might produce a catchment response to climate warming indicative of an ‘adaptive capacity’ of the forest (Gunderson, 2000).

In this study, we examined changes in a catchment's DI and EI coincident with climatic transitions from relatively cool to warm conditions. We looked specifically for deviations from the Budyko curve with time to determine whether the catchments shifted predictably in terms of their water balance. To that end, we developed quantitative metrics to express changes in a catchment's Budyko characteristics with time. *Dynamic deviation* (*d*) is a measure of change in a catchment's EI relative to the Budyko curve as climate varies – in other words, a measure of the extent to which the allocation of precipitation to ET vs. runoff matches theoretical expectations. *Elasticity* is a measure of a catchment's ability to maintain water partitioning consistent with the Budyko curve as climate varies (i.e., the ratio of a catchment's range in DI to its range in EI). Elasticity of water yield to changes in *P* has shown utility in quantifying hydrological sensitivity to climate change (Schaake, 1990; Sankarasubramanian *et al.*, 2001); we apply elasticity to Budyko characteristics. A catchment has high elasticity if its DI changes with climate warming, but EI changes only slightly. In contrast, a catchment has low elasticity if EI responds substantially to changes in DI.

We used elasticity as an indicator of the hydrological resilience of catchments. Hydrological resilience is exhibited when a change in DI results in a corresponding change in EI such that the system moves along the theoretical Budyko curve – i.e., its water yields respond consistently with theoretical expectations (high elasticity and low deviation). A lack of hydrological resilience is exhibited when a change in DI results in a corresponding change in EI that pushes the system away from the theoretical Budyko curve – i.e., its water yields are larger or smaller than would be predicted from theoretical expectations (low elasticity and high deviation). A nonresilient state could lead to fundamental changes in forest structure and function and possibly shift the catchment into a permanent alternative state.

We investigated how water partitioning between ET and runoff has responded over time to climate warming in forested headwater systems, and how forest type and forest history affect hydrological resilience to climate warming. In answering this question, we tested two hypotheses. First, during climate warming, resilient catchments (high elasticity and low deviation) will shift along the Budyko curve, but nonresilient catchments (low elasticity and high deviation) will deviate upward from the theoretical curve, indicating a decrease in water yield. The magnitude of decline in water yields (increasing EI) will be a positive function of the extent of warming but may be modified by the direction of precipitation change. Wetter conditions serve as a negative feedback (less deviation), while drier conditions serve as a positive feedback (more deviation). Second, elastic catchments will be characterized by relatively undisturbed conditions, with mixed forests being more elastic than either purely coniferous or deciduous forests and with older forests being more elastic than younger forests (recognizing that we may not have sufficient sample size to test the role of forest age as rigorously as we would like). The relatively short cool and warm periods used in this study (5 years) give us a basic understanding of catchment responses to changing climate, which can then give us an indication of what longer-term responses might be.

Our analysis uses long-term monitoring data from headwater catchments, including sites of the United States (US) Long Term Ecological Research (LTER), US Forest Service, US Geological Survey, and Canadian HydroEcological Landscape Processes (HELP) networks. Each site benefits from a generation or more of site studies of local processes and patterns. This analysis is one of the first to combine US and Canadian data from coast to coast to explore headwater catchment responses to changing environmental conditions across broad climatic gradients.

## Materials and methods

### Study sites

**Fig 2:**
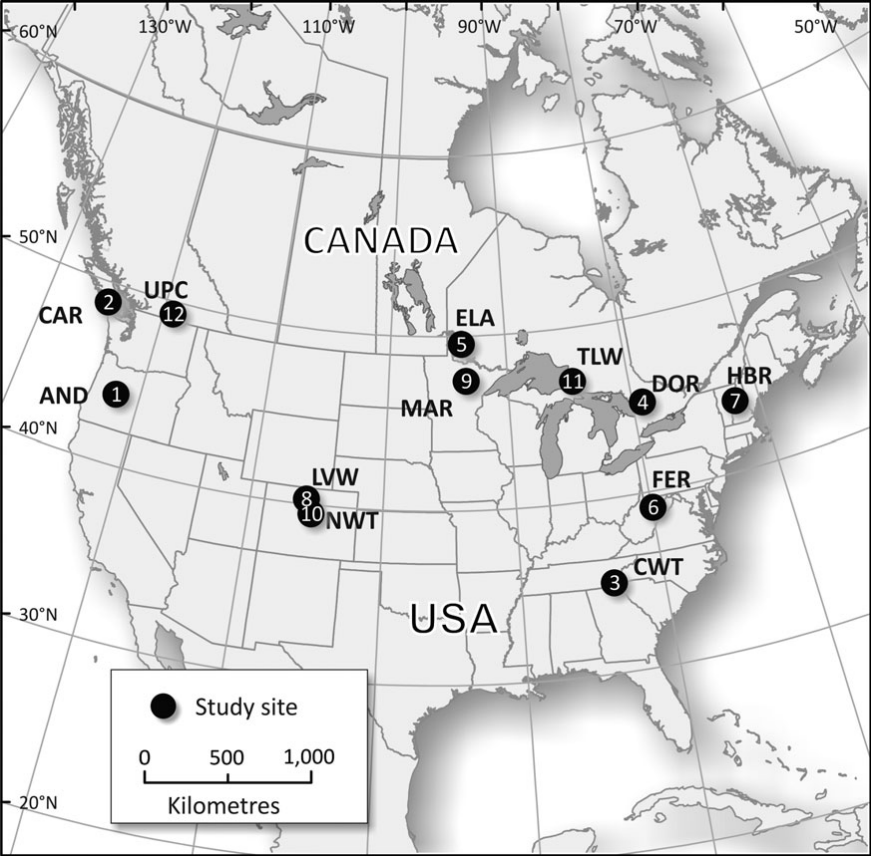
Location of long-term monitoring catchments that met the selection criteria for this study (*n *=* *12). Site identifiers are: 1, HJ Andrews; 2, Carnation; 3, Coweeta; 4, Dorset; 5, Experimental Lakes Area; 6, Fernow; 7, Hubbard Brook; 8, Loch Vale Watershed; 9, Marcell; 10, Niwot; 11, Turkey Lakes Watershed; 12, Upper Penticton.

More than 100 potential catchments from the combined networks were examined as possible candidates for the analysis of catchment response to climate warming. We selected forested and alpine headwater catchments that were located within forest regions that had (a) no anthropogenic disturbances since 1950; (b) a minimum of 15 years since 1980 of consecutive and coincident records of daily air temperature (*T*, °C), precipitation (*P*, mm yr^−1^), and water yield (*Q*, L s^−1^); and (c) detectable shifts from cooler to warmer air temperatures. These criteria resulted in the selection of 21 headwater catchments at 12 sites (Fig.2; Tables1 and 2; Table S1). At some sites, multiple catchments were selected if they provided a contrast in catchment properties that could influence water partitioning. While these criteria resulted in a relatively small sample size and limits the detail of the analysis, there is enough variety in geographic area and site characteristics to make general observations about the effects of climate warming on different forest types and ages.

**Table 1:** Description of catchments used in the Budyko curve analysis

ID	Site	Catchment code	Catchment name	Area (ha)	Dominant species	Soils and geomorphology	Bedrock geology
1a	HJ Andrews	AND 2	WS02	60	Douglas fir and western hemlock	Holocene; steep (>30°) planar slopes with thin (1–2 m) soil; slump benches and head scarps	Miocene volcanic breccia and sedimentary rocks capped by lava flows
1b	HJ Andrews	AND 8	WS08	21	Douglas fir and western hemlock	Holocene; Moderate (6–10°) slopes with thick soil (2+ m); irregular landslide terrain	Miocene volcanic breccias and lava flows
2	Carnation	CAR	Sub-watershed WS C	146	Western hemlock, western red cedar, Amabilis fir, old growth	Mixture of morainal veneer, colluvial veneer, and morainal blanket with minor rock outcrops	Jurassic volcanics of the Bonanza group consisting of basaltic to rhyolitic lava, tuff, beccia, minor argillite and graywacke, and Island intrusives consisting of granodiorite, quartdiorite, granite and quartz monzonite
3a	Coweeta	CWT 17	Watershed 17	14	Eastern white pine plantation	Holocene to Tertiary; Colluvial sediments, discontinuous; Discontinuous, or patchy in distribution; soils are in the Saunook series, a fine-loamy, mixed, mesic Humic Hapludult, found at streamside positions, and Cowee-Evard complex soils, fine-loamy, mixed-oxidic, mesic, Typic Hapludult, found on ridge positions	Basal coarse-grained quartz diorite gneiss (Persimmon Creek Gneiss), overlain with metasandstone and politic schist (Coleman River Formation), overlain by quartzose metasandstone and quartzite (Ridgepole Mountain Formation)
3b	Coweeta	CWT 18	Watershed 18	13	Mixed oak hardwood	Holocene to Tertiary; Colluvial sediments, discontinuous; Discontinuous, or patchy in distribution; soils are in the Saunook series, a fine-loamy, mixed, mesic Humic Hapludult, found at streamside positions, and Cowee-Evard complex soils, fine-loamy, mixed-oxidic, mesic, Typic Hapludult, found on ridge positions	Basal coarse-grained quartz diorite gneiss (Persimmon Creek Gneiss), overlain with metasandstone and politic schist (Coleman River Formation), overlain by quartzose metasandstone and quartzite (Ridgepole Mountain Formation)
4a	Dorset	DOR HP3	Harp Lake 3	26	Sugar maple and red maple with some beech, birch, and hemlock; wetland areas dominated by black spruce	Till Veneer, thin and discontinuous till; may include extensive areas of rock outcrop; Coarse grained (Glacio)Lacustrine, sand, silt, and gravel; deposited as deltas, sheet sands, and lag deposits	Precambrian; early Mesoproterozoic metamorphic rocks; orthogneiss
4b	Dorset	DOR HP 3A	Harp Lake 3A	20	Sugar maple and red maple with some beech, birch, and hemlock; wetland areas dominated by black spruce	Till Veneer, thin and discontinuous till; may include extensive areas of rock outcrop; Coarse grained (Glacio)Lacustrine, sand, silt, and gravel; deposited as deltas, sheet sands, and lag deposits	Precambrian; early Mesoproterozoic metamorphic rocks; orthogneiss
4c	Dorset	DOR HP 4	Harp Lake 4	123	Sugar maple and red maple with some beech, birch, and hemlock; wetland areas dominated by black spruce	Thin (1–10 m thick) veneer of discontinuous till with extensive areas of rock outcrop; Coarse grained (Glacio)Lacustrine, sand, silt, and gravel; deposited as deltas, sheet sands, and lag deposits	Precambrian; early Mesoproterozoic metamorphic rocks; granitized biotite and hornblende gneiss
4d	Dorset	DOR HP 5	Harp Lake 5	191	Sugar maple and red maple with some beech, birch, and hemlock; wetland areas dominated by black spruce	Till Veneer, thin and discontinuous till; may include extensive areas of rock outcrop; Coarse grained (Glacio)Lacustrine, sand, silt, and gravel; deposited as deltas, sheet sands, and lag deposits	Precambrian; early Mesoproterozoic metamorphic rocks; granitized biotite and hornblende gneiss
4e	Dorset	DOR PC	Plastic Lake	27	White pine, eastern hemlock and red maple	Till Veneer, thin and discontinuous till; may include extensive areas of rock outcrop	Precambrian; early Mesoproterozoic metamorphic rocks; granitized biotite and hornblende gneiss
5	Experimental Lakes Area	ELA	Watershed 239	400	Jackpine and black spruce	Till Veneer, thin and discontinuous till; may include extensive areas of rock outcrop; (Glacio)Lacustrine acidic brunisol, silt loam soils	Precambrian; undivided Neoarchean intrusive rocks and undivided granitoid rocks
6	Fernow	FER	Watershed 4	39	Oak-hickory forest	Steep slopes (20–40%), with thin soils (<1 m); Colluvial sediments, discontinuous	Paleozoic Devonian; predominantly interbedded sandstones and shale, some marine sediment layers outcropping
7a	Hubbard Brook	HBR 3	Watershed 3	42	Sugar maple, beech and yellow birch	Pleistocene; late Wisconsinan; glacial till, mostly sandy loam; thickness ranges from 0 m at bedrock outcrops on the upper watershed border to over 5 m thick	Paleozoic Silurian; mica schist, quartzite and calc-silicate granulite
7b	Hubbard Brook	HBR 6	Watershed 6	13	Sugar maple, beech and yellow birch	Pleistocene; late Wisconsinan; glacial till, mostly sandy loam; thickness ranges from 0 m at bedrock outcrops on the upper watershed border to over 5 m thick	Paleozoic Silurian; mica schist, quartzite and calc-silicate granulite
8	Loch Vale	LVW	Andrews Creek	183	Alpine tundra	Holocene till, talus, and colluvium; Discontinuous, or patchy in distribution	Precambrian granitic and metamorphic rocks
9a	Marcell	MAR 2	Watershed S2	10	Aspen, birch, black spruce	Pleistocene; late Wisconsinan to pre-Illinoian; Glacial till over outwash sands, mostly silty, thick; 50 m	Early Precambrian granitic rocks
9b	Marcell	MAR 5	Watershed S5	53	Aspen, birch, black spruce	Pleistocene; late Wisconsinan to pre-Illinoian; Glacial till over outwash sands, mostly silty, thick; 50 m	Early Precambrian granitic rocks
10	Niwot	NWT	Upper Green Lakes (GL4)	225	Alpine tundra	Holocene; accumulated since deglaciation about 12 000 years ago	Precambrian schists and gneisses, the Silver Plume quartz monzonite
11a	Turkey Lakes	TLW 35	Catchment c35	4	Sugar maple	Till Veneer, generally thin (<2 m) with areas of rock outcrop at higher elevations and steeper slopes	Precambrian; silicate greenstone with small outcrops of more felsic igneous rocks
11b	Turkey Lakes	TLW 38	Catchment c38	6	Sugar maple	Till Veneer, generally thin (<2 m) with areas of rock outcrop at higher elevations and steeper slopes	Precambrian; silicate greenstone with small outcrops of more felsic igneous rocks
12	Upper Penticton	UPC	Two Forty Creek	500	Lodgepole pine	Till mantle with minor glaciofluvial sands and gravels, includes extensive areas of rock outcrop at higher elevations	Cretaceous or Jurassic Okanagan Batholith; massive, medium-coarse-grained, light gray biotite granodiorite and granites

**Table 2:** Catchment 5-water-year (5-wyr) cool periods (period with lowest average temperature) and 5-wyr warm periods (period with highest average temperature), changes in temperature and precipitation during shift from cool to warm period, as well as components of catchment departures from the Budyko curve [static (*s*) and dynamic (*d*) deviations] and catchment abilities to maintain water partitioning consistent with the Budyko curve as climate varies (elasticity *e*). Catchment ecosystem type (alpine, coniferous, deciduous or mixed coniferous and deciduous forest) and age also provided

ID	Catchment	Cool period	Warm period	Δ*T* (°C)	Δ*P* (%)	*s*	*d*	*e*	Forest type	Forest age (years)
1a	AND 2	1982–1986	1988–1992	0.57	−21	0.16	−0.01	1.61	Coniferous	450–500
1b	AND 8	1982–1986	1988–1992	0.57	−21	0.19	0.03	1.33	Coniferous	450–500
2	CAR	1985–1989	1990–1994	0.43	9	0.07	−0.18	0.23	Coniferous	>100
3a	CWT 17	1977–1981	1989–1993	1.13	13	0.17	0.02	2.08	Coniferous	60
3b	CWT 18	1977–1981	1989–1993	1.13	13	−0.04	0.01	1.61	Deciduous	80
4a	DOR HP3	1992–1996	1998–2002	1.65	−12	−0.04	0.04	1.04	Deciduous	>100
4b	DOR HP 3A	1992–1996	1998–2002	1.65	−12	−0.02	0.00	1.20	Deciduous	>100
4c	DOR HP 4	1992–1996	1998–2002	1.65	−12	−0.02	0.05	0.83	Deciduous	>100
4d	DOR HP 5	1992–1996	1998–2002	1.65	−12	−0.07	0.08	0.66	Deciduous	>100
4e	DOR PC	1992–1996	1998–2002	1.81	−8	−0.04	0.00	0.98	Mixed	>100
5	ELA	1993–1997	1998–2002	1.85	14	0.09	−0.01	1.68	Coniferous	>100
6	FER	1977–1981	1987–1991	1.44	−6	0.11	−0.02	1.24	Deciduous	90–100
7a	HBR 3	1992–1996	1998–2002	1.36	−4	−0.04	0.00	1.98	Deciduous	100
7b	HBR 6	1992–1996	1998–2002	1.36	−4	−0.03	−0.02	2.09	Deciduous	100
8	LVW	1995–1999	2000–2004	0.88	−27	0.04	−0.17	0.35	Alpine	>100
9a	MAR 2	1993–1997	1998–2002	2.12	−2	0.22	–0.05	2.91	Mixed	>80
9b	MAR 5	1993–1997	1998–2002	2.91	−2	0.31	−0.05	2.72	Mixed	>80
10	NWT	1992–1996	2000–2004	0.67	−17	0.20	−0.16	0.33	Alpine	>100
11a	TLW 35	1992–1996	1998–2002	1.95	−12	0.11	0.01	1.16	Deciduous	>140
11b	TLW 38	1992–1996	1998–2002	1.95	−12	0.14	−0.05	1.51	Deciduous	>140
12	UPC	1995–1999	2002–2006	0.59	−13	0.04	−0.08	0.72	Coniferous	125

### Dryness index (DI) and Evaporative index (EI)

For each catchment, *T*, *P*, and *Q* data were converted from daily to average monthly and annual *T* and total monthly and annual *P* and *Q* values (over water years, October through September). For sites with multiple *T* or *P* stations, the recommendations of local site researchers were followed in choosing either a representative single station record or some combination of the multiple station records.

Water-year PET was calculated for each catchment as a function of average monthly *T* according to the Hamon (1963) formula because only *T* data were available for all sites. The Hamon formula has a tendency to underestimate PET (Yao, 2009), but performs better than other *T*-based PET models and is comparable to common radiation-based PET models (Lu *et al.*, 2005). Water-year AET was estimated using a water balance approach and measurements of annual *P* and *Q*: AET = *P* − *Q* − Δ*S*, where Δ*S* is change in water storage volume. We assumed steady-state water storage (i.e., Δ*S* = 0) for the time periods encompassed in this study. Both PET and AET estimates may be affected by variation in groundwater recharge and storage among sites due to different surficial and bedrock geologies (Table1).

### Budyko curve

The Budyko curve was developed as a theoretical expression to explain how annual water balance is partitioned as a function of the relative magnitude of water and energy supply. Several attempts have been made to derive theoretical equations that explain this relationship, and these equations have been applied and modified for catchments around the world. We used the equation from Zhang *et al*. (2001), which accounts for plant-available water *w* that was tailored specifically for different catchments (i.e., *w *=* *2 in forested catchments, *w *=* *0.5 in grassland or cropland catchments, and *w *=* *1 in mixed cover catchments). We used the Zhang *et al*. (2001) model to give the theoretical relationship between DI and EI in our catchments using *w *=* *2 for all catchments.

### Climate warming shifts

For each catchment, a 5-water-year (5-wyr) moving average of the *T* time series was calculated. A catchment's ‘cool period’ was defined as the 5-wyr period with the minimum 5-wyr *T*. A catchment's ‘warm period’ was defined as the first 5-wyr period after the cool period (no overlapping years) for which the 5-wyr *T* was (a) warmer than the previous 5-wyr *T* and (b) warmer than the subsequent three 5-wyr (moving-average) *T* values by more than 1 standard deviation. All such warming shifts were identified in the *T* record, and the largest shift was then selected as the basis for this analysis. The ‘break point’ is the last year of the designated cool period. The selected cool and warm periods did not necessarily include the temperature minima and maxima observed during the periods of record (Table2).

### Budyko metrics: deviation and elasticity

We developed several custom indices to describe the potential departure from the theoretical Budyko curve of a catchment's DI and EI points with time.

**Fig 3:**
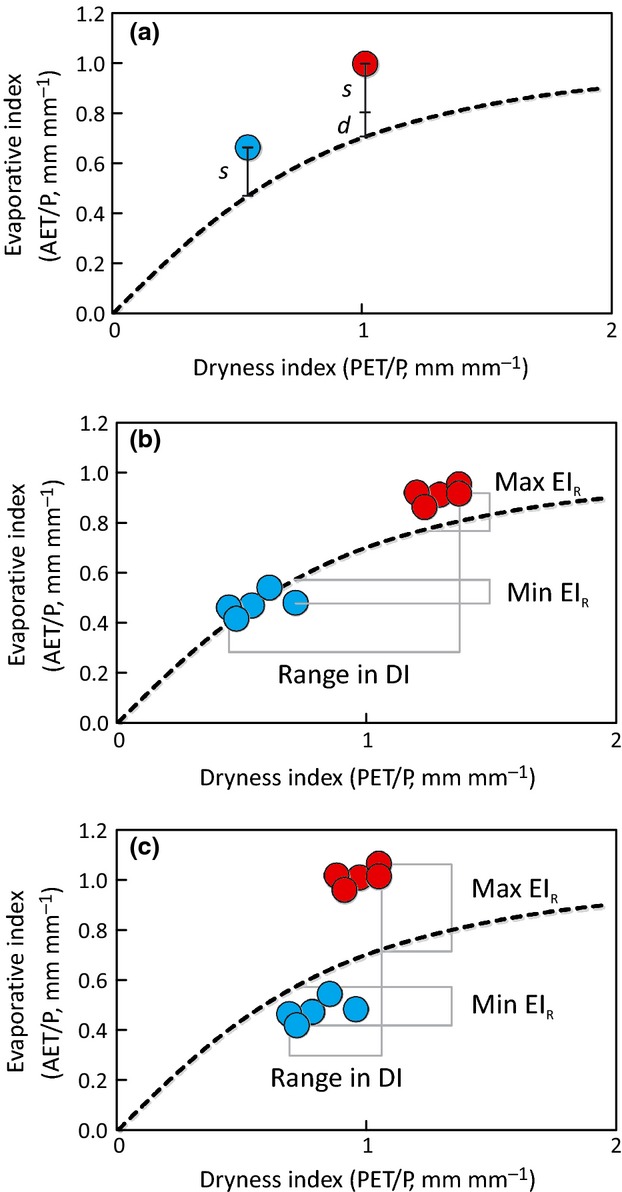
Graphical representation of Budyko resilience metrics. Each dot shows a catchment's paired dryness index (DI) and evaporative index (EI) values: blue for the cool period and red for the later warm period. The dashed line represents the theoretical Budyko curve. (a) Static deviation (*s*) was calculated as the difference between measurement-based and theoretical evaporative indices during the catchment's cool period: *s* = EI_M,cool_ − EI_B,cool_. Dynamic deviation (*d*) was calculated as the analogous warm-period quantity, corrected for the previously determined *s*: *d* = EI_M,warm_ − EI_B,warm_ − *s*. Points that fall above the theoretical curve indicate smaller-than-predicted water yields; points that fall below the curve indicate larger-than-predicted yields. Elasticity (*e*) was calculated as the ratio of a catchment's range in DI to its range in EI during the two contrasting climate periods: *e *= (DI_max_ − DI_min_)/(EI_R,max_ − EI_R,min_). (b) This example catchment exhibited a high degree of elasticity (*e *>* *1) (i.e., approximating theoretical behavior). (c) This example catchment exhibited low elasticity (*e *<* *1) (i.e., deviating from theoretical behavior).

*Deviation* was characterized as a vertical departure from the Budyko curve – i.e., the difference between a catchment's measured EI (EI_M_) and its theoretical value (EI_B_, predicted as a function of DI according to the Budyko curve). Two components of deviation were calculated. *Static deviation* (*s*) results from inherent catchment characteristics that are assumed to be constant with time. *Dynamic deviation* (*d*) results from catchment changes over time – in this case, in response to climatic warming. Static deviation for each catchment was based on the cool-period observations; i.e., *s *= EI_M,cool_ − EI_B,cool_ (Fig.3a). Dynamic deviation was considered to be that portion of warm-period deviation, corrected for this static component; i.e., *d *= EI_M,warm_ − EI_B,warm_ − *s* (Fig.3a).

*Elasticity* (*e*) was calculated as the ratio of the range in water-year DI values to the range in water-year EI residual values experienced during the period encompassing the identified cool and warm periods; i.e., *e *= (DI_max_ − DI_min_)/(EI_R,max_ − EI_R,min_) (Fig.3b, c). The DI : EI relationship changes when moving right along the theoretical Budyko curve. We accounted for this by using the residuals of the EI values (EI_R_) for each year for the period of record (EI_R_ = EI_M_ − EI_B_) to calculate *e*. A catchment with high elasticity partitions *P* into *Q* and ET in a manner that produces smaller changes in EI_R_ values relative to changes in DI values and therefore varies predictably with the Budyko curve (Fig3b). A catchment with low elasticity partitions water in a less predictable manner (Fig.3c). We used *e *=* *1 as the defining threshold for elastic vs. inelastic catchments.

### Warming with precipitation feedbacks

Shifts to warmer conditions were often accompanied by a change in precipitation (Δ*P*). To elucidate potential interactions among Δ*T*, Δ*P*, *d*, and *e*, we classified catchments based on both the degree of warming (i.e., the magnitude of Δ*T*) and the degree of drying or wetting (i.e., the magnitude of negative or positive Δ*P*). Data for any year following an extreme annual *P* occurrence (defined as >1.5 standard deviations from the long-term mean annual *P*) were removed because extreme *P* years resulted in ‘legacy effects’ that amplified *d* of the following year. Catchments were classified into one of six different climate-shift categories, first by dividing Δ*T* into two categories according to whether the catchments experienced relatively little warming (Δ*T* < 1.5 °C) or greater warming (Δ*T* > 1.5 °C). Catchments were further subdivided according to whether the catchments became appreciably wetter (Δ*P* > 10%), experienced relatively little change (−10% < Δ*P* < 10%), or became appreciably drier (Δ*P* < −10%). Deviations from the Budyko curve as a function of both warming (and associated wetting or drying) and elasticity were examined by conducting regression analyses using spss version 20.0. (IBM Corp., Armonk, NY, USA).

## Results

### Static deviations inherent during cool period

**Fig 4:**
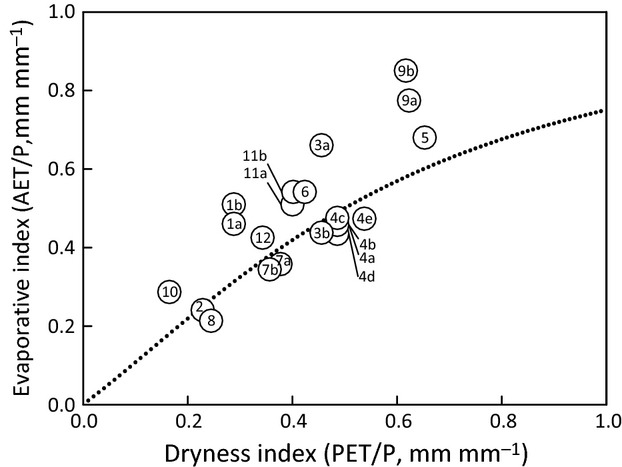
Mean annual dryness index and evaporative index values for headwater catchments during the 5-water-year cool period. The dotted line represents the Zhang *et al*. (2001) modification of the Budyko curve (*w *=* *2). The vertical displacement of each point from the Budyko curve is the static deviation *s*. Key to site IDs (the numbers within the circles) is given in Table1.

Static deviation (*s*) describes the vertical displacement of a 5-wyr cool-period (DI, EI) point from the theoretical Budyko curve caused by inherent characteristics of a catchment (Fig.4; Table2). Vertical deviations from the Budyko curve ranged from −0.07 to 0.31 (Table2). Catchments with *s *<* *0 exhibited prewarming water yields that were higher than expected based on Budyko's theoretical predictions; catchments with *s *>* *0 exhibited lower water yields than expected. Catchment points falling in close proximity to the curve (|*s*| < 0.05) indicated prewarming water yields that were consistent with the theoretical predictions of the Budyko curve. For the eight catchments that fell below the curve, the magnitude of *s* was small (range of −0.02 to −0.07), indicative of water yields marginally greater than expected. In contrast, for the 13 catchments that fell above the curve, the magnitude of *s* was comparatively large (range of 0.04–0.31), indicative of water yields marginally to substantially smaller than expected (Table2). Local experts at some sites assisted with the identification of factors that may have influenced *s*, including forest disturbance legacies, surface storage mechanisms, surface water/ground water interactions, as well as imperfect measurement or inadequate characterization of *P*, *T*, or *Q* in the catchment (Table S1).

### Dynamic deviation coincident with warming

**Fig 5:**
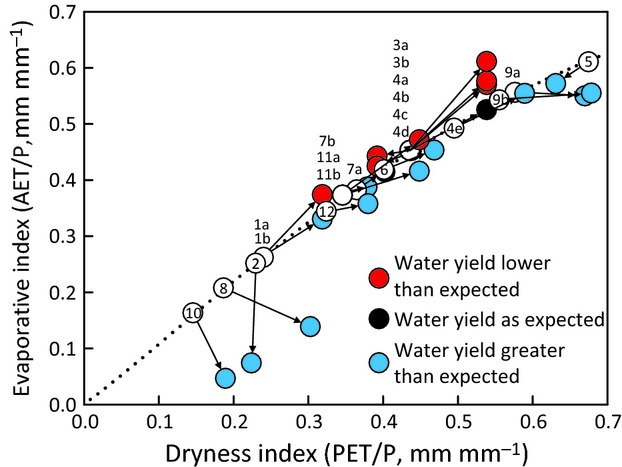
Mean cool-period and warm-period dryness index (DI) and evaporative index (EI) values for headwater catchments showing catchment transitions from 5-water-year (5-wyr) cool period (numbered circles) to 5-wyr warm period (colored circles) with static deviation (*s*) removed from both periods. Arrows denote the direction of movement from cool to warm period. Red circles denote catchments with decreases in expected water yield (increasing EI); blue circles denote catchments with increases in expected water yield (decreasing EI); and black circles denote catchments with expected water yield. The dotted line represents the Zhang *et al*. (2001) modification of the Budyko framework (*w *=* *2). Key to site IDs (the numbers within the circles) is given in Table1.

Dynamic deviation (*d*) is given by the vertical departure of the 5-wyr warm-period (DI, EI) point from the Budyko curve once *s* has been removed (Fig.5). Of the 21 catchments, 11 had warm-period water yields greater than predicted by the Budyko relation (*d *<* *0), three had warm-period water yields that were as expected (*d *=* *0), and seven had warm-period water yields smaller than expected (*d *>* *0). Values of dynamic deviation ranged from *d = *−0.18 (below the curve) to *d = *0.08 (above the curve) (Table2). For catchments below the curve, the magnitudes of dynamic deviation were often larger (range of *d *= −0.18 to −0.01), indicating relatively larger increases in water yield (Table2). For catchments above the curve, the magnitudes of dynamic deviation were smaller (range of *d *=* *0.01–0.08), indicating a smaller range of decreases in water yield (Table2). No obvious patterns emerged in terms of why a specific catchment's water yield would respond with a negative, neutral, or positive response to climate warming (Table1).

### Elasticity

**Fig 6:**
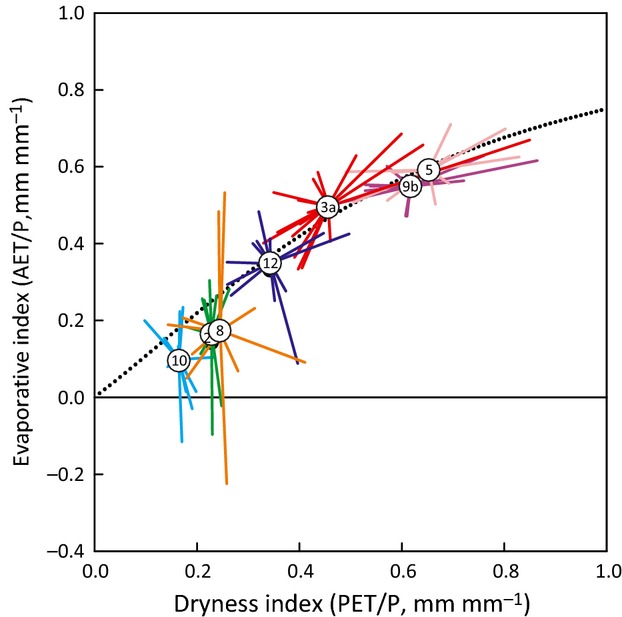
Year-to-year variability in mean annual dryness index and evaporative index values for selected headwater catchments during period of record with static deviation (*s*) removed from each value. The numbered circles represent the mean annual values over the period of record. The radiating lines indicate annual excursions from that mean. The longer the line, the greater the departure from the long-term mean value. The dotted line represents the Zhang *et al*. (2001) modification of the Budyko framework (*w *=* *2). Key to site IDs (the numbers within the circles) is given in Table1.

Figure6 shows the interannual variability in DI and EI points for representative catchments for the period of record. Elasticity (*e*) ranged from 0.23 to 2.91 (Table2). Seven catchments exhibited a broad range in EI but not DI [i.e., vertical variation dominated, yielding a low elasticity (*e *<* *1)]; the remaining 14 catchments exhibited a broad range in DI but not EI [i.e., horizontal variation dominated, yielding a high elasticity (*e *>* *1)]. Catchments ELA (ID #5) and MAR (ID #9b) exhibited relatively high DI and tended to show broad interannual ranges in DI but not EI (Fig.6). Catchments CAR (ID #2), LVW (ID #8) and NWT (ID #10), in contrast, exhibited relatively low DI and tended to show broad interannual ranges in EI but not DI (Fig.6). At intermediate DI values, both patterns of interannual variability were found.

### Budyko metrics vs. dynamic deviation

Our first hypothesis was that elastic catchments (*e *>* *1, our metric for resilience) would shift along the Budyko curve under warming conditions, but that inelastic catchments (*e *<* *1, our metric for nonresilience) would deviate away from it. We predicted that inelastic catchments would deviate upward from the theoretical curve, indicating a decrease in water yield coincident with warming. We also predicted that the magnitude of this deviation would be a positive function of the degree of warming, but that wetter conditions would serve as a negative feedback (leading to less deviation), while drier conditions would serve as a positive feedback (leading to more deviation).

**Fig 7:**
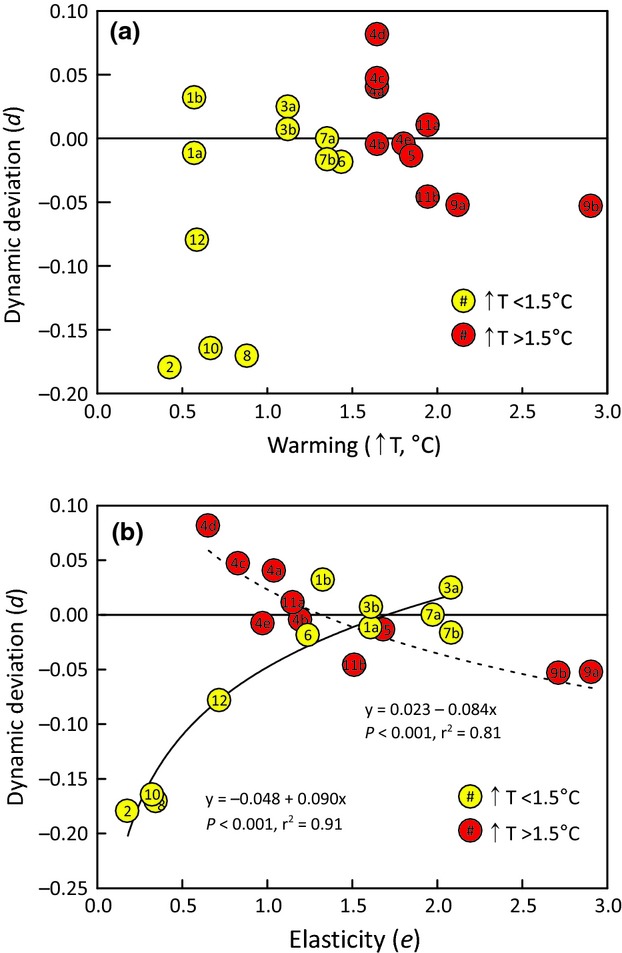
Dynamic deviations of headwater catchments as a function of (a) warming and (b) elasticity. The color of the circle represents the extent of warming over the cool-to-warm transition (yellow = <1.5 °C warming; red = >1.5 °C warming). The long-dash line in (b) represents the relationship between *d* and *e* for catchments that experienced <1.5 °C warming. The short-dash line in (b) represents the relationship between *d* and *e* for catchments that experienced >1.5 °C warming. Key to site IDs (the numbers within the circles) is given in Table1.

Dynamic deviation in water yield during the cool-to-warm climate shift was not explained by the degree of warming (Fig.7a). Wetter conditions could conceivably counterbalance the effects of warmer temperatures, but when we removed from consideration those catchments where Δ*P* > 10% [i.e., CWT 17 (ID #3a), CWT 18 (ID #3b), and ELA (ID #5)], dynamic deviation was still not explained by the extent of warming (data not shown).

Dynamic deviation in water yield during the cool-to-warm climate shift varied with elasticity (Fig.7b). Catchments with relatively low elasticity (*e *<* *1) were more likely to experience a negative deviation (increase in water yield) in response to warming (*r*^2^ = 0.34, *P *<* *0.01; line not shown). However, when we classified the catchments into two rates of warming (Δ*T* < 1.5 °C and Δ*T* > 1.5 °C), stronger relationships emerged. Catchments that experienced a relatively small degree of warming (Δ*T* < 1.5 °C; yellow circles in Fig.7) showed a significant exponential decrease in dynamic deviation as elasticity declined (*r*^2^ = 0.91, *P *<* *0.001). In contrast, catchments that experienced relatively high rates of warming (Δ*T* > 1.5 °C; red circles) showed a significant exponential increase in dynamic deviation as elasticity declined (*r*^2^ = 0.81, *P *<* *0.001). For catchments with low elasticity (*e *<* *1), the relationships between elasticity and dynamic deviation exhibited slopes of different signs, depending on the degree of warming (Fig.7b). Classifying catchments according to whether they became appreciably wetter (Δ*P* > 10%), experienced relatively little change (−10% < Δ*P* < 10%), or became appreciably drier (Δ*P* < −10%), did not have an effect on the relationship between dynamic deviation and either warming or elasticity (data not shown).

### Influence of forest type and age on elasticity

Our second hypothesis was that elastic catchments were characterized by forests that contained a diversity of forest types and ages, and that EI reflected the capacity of the ecosystem to adapt to changing climatic conditions. We predicted that mixed forests would be more elastic than either coniferous or deciduous forests. We also predicted that older forests would be more elastic than younger ones.

**Fig 8:**
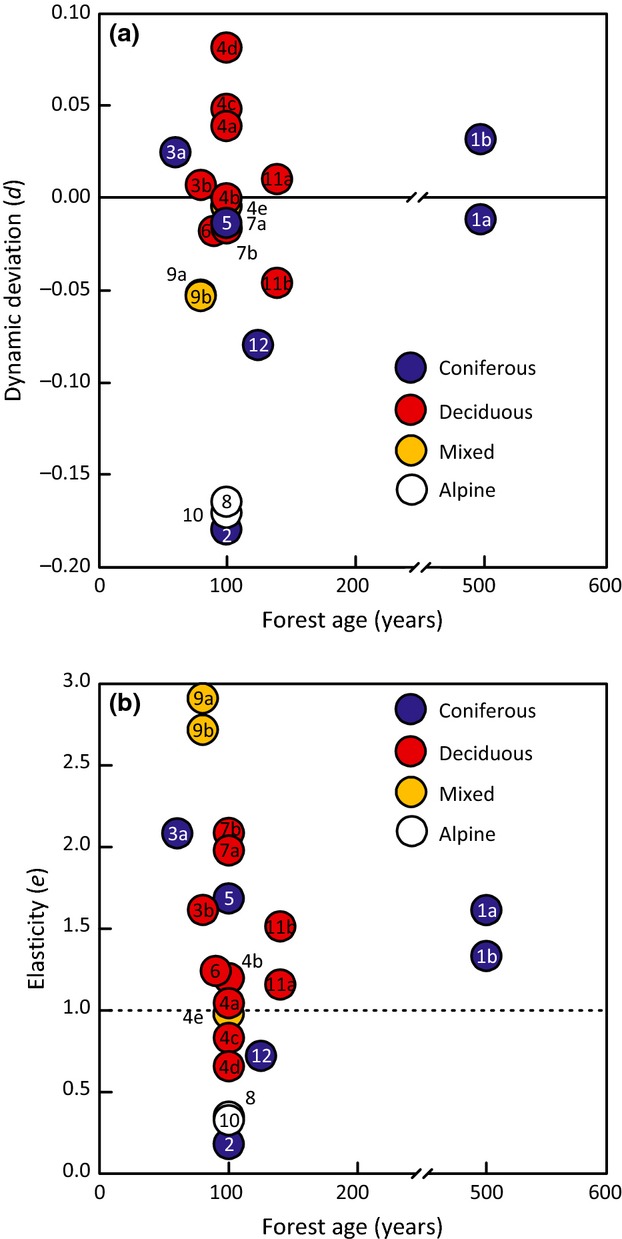
Catchment properties as a function of forest type (colored circles) and forest age: (a) dynamic deviation *d* and (b) elasticity *e*. Key to site IDs (the numbers within the circles) is given in Table1.

In our data set, dynamic deviation varied among forest types and perhaps forest ages (Table2; Fig.8). The alpine catchments (IDs # 8 and #10) experienced small increases in *T* (Δ*T* < 1.5 °C) and large (>10%) decreases in *P* (Table2; Fig.7). Elasticity was low (*e *<* *0.5) and dynamic deviation was substantial and negative (*d *< −0.15). These catchments had larger-than-expected water-yield increases associated with warming, perhaps due to glacier or permafrost melt.

Conifer catchments were generally situated in western North America and experienced slight warming (mostly Δ*T* < 1 °C, with the exception of CWT17 (ID #3a) and ELA (ID #5), the two conifer catchments that were situated in eastern North America, which experienced Δ*T* > 1 °C) with either decreases or increases in *P* (Table2). They had a wide range of elasticity (*e *<* *0.5–2.0) and wide-ranging but mostly negative dynamic deviation (*d *= −0.2 to 0.0). Those with the lowest elasticity [CAR (ID #2) and UPC (ID #12)] had the most negative dynamic deviation with larger-than-expected water yields. In contrast, those with greater elasticity (*e *>* *1) had near-zero dynamic deviations (no change in water yields).

The deciduous catchments were all situated in eastern North America and experienced intermediate increases in *T* (1–2 °C) with either decreases or increases in *P* (Table2). They had a slightly narrower range of elasticity (*e *=* *0.5–2.0), and near-zero to mostly positive dynamic deviation (*d *= −0.05–0.1). Those with the lowest elasticity [DOR HP3 (ID #4a), HP4 (ID #4c), and HP5 (ID #4d)] had the highest positive dynamic deviation with smaller-than-expected water yields. The one exception was TLW38 (ID #12b), a sugar maple forest in the Turkey Lakes Watershed of central Ontario (*d *= −0.05). Some 20% of this catchment area is wetland, which may have provided a water supply to sustain water yields when climate shifted to warmer conditions.

The mixed deciduous-conifer forest sites, which were all situated in eastern North America, experienced the largest changes in *T* (mostly Δ*T* > 2 °C) and also decreasing *P* (Table2). These exhibited a wide range of elasticity, including sites with the highest elasticity (*e *=* *1.0–3.0) and slightly negative to near-zero dynamic deviation (*d *= −0.05 to 0). Catchments with this type of forest stayed the closest to the Budyko curve despite experiencing the greatest climate warming.

The range of forest ages among our sites was admittedly limited (Table2, Fig.8). This is partly due to our selection criteria, which required undisturbed forest since 1950 (older forests were often disturbed) and to a general lack of experimental catchments with older forests. However, there is a suggestion of convergence in dynamic deviation values to near zero and convergence of elasticity toward 1 with forest age (Fig.8a, b). The magnitude of dynamic deviation (positive or negative) was closest to zero and elasticity was closest to 1 for the two catchments with the oldest forests [AND2 (ID #1a) and AND8 (ID #1b), which were 450–500 years in age].

## Discussion

Climate change is expected to affect forest water yields (Aber *et al.*, 1995). However, not all forest ecosystems are expected to respond in a uniform manner. Rates of climate change vary geographically (Walther *et al.*, 2002; Karl *et al.*, 2009; Loarie *et al.*, 2009), and forests of different types and ages may influence catchment responses (Brown *et al.*, 2005; Ewers *et al.*, 2005). The results of our study investigating the responses of forested catchments to relatively short-term transitions from cool to warm conditions provide a conceptual basis for understanding and predicting the direction and magnitude of forest headwater yield response to climate change.

Ponce Campos *et al*. (2013) observed that the water-use efficiency (the ratio of above-ground net primary production to ET) in forests was sensitive to water availability. Higher water-use efficiencies were observed in drier years, and lower (native) water-use efficiencies were observed in wetter years. This flexibility in water-use efficiency suggests a resilience of the ecosystem to climate variability and in particular to climatic extremes observed in recent decades. Holling (1973, 1996) identified two distinct resilience concepts – engineering and ecological resilience. The hydrological responses of our headwater catchments exhibited engineering resilience because they hovered around an attractor state (mapped in EI vs. DI space), occasionally deviating from the attractor state defined by the Budyko curve (not necessary along the curve) due to a climatic variability or climatic extremes but ultimately returning to the Budyko curve. An ecological resilience would have occurred if, for example, the vegetation resisted change or if the vegetation community composition changed and shifted the weighted average stomatal conductance. We do not think we have evidence of ecological resilience in the data presented in this study. Ponce Campos *et al*. (2013) urged that the development of a predictive understanding of climatic threshold beyond which resilience will break down is needed to predict consequences of anticipated future climate change on water yields.

We used elasticity as a metric for resilience. We hypothesized that elastic catchments (*e *>* *1) would shift along the Budyko curve and that inelastic catchments (*e *<* *1) would deviate upward from the curve, yielding less water than predicted by the theoretical relationship between DI and EI. We also hypothesized that elastic catchments would have a diversity of forest types and ages such that they would have the capacity to adapt to changing climatic conditions and therefore would have small changes in EI. We found that different forest types responded differently to climate warming. Catchments with high elasticity experienced little to no changes in water yields, whereas catchments with low elasticity experienced unpredictably larger or smaller water yields.

Our results are distinct from recent papers that use a Budyko curve approach to examine climate change and its influence on water yield (e.g., Wang & Hejazi, 2011; Williams *et al.*, 2012; Troch *et al.*, 2013). We used existing empirical datasets from forested headwater catchments that were not affected by land cover or land use changes to draw inferences about how forest type and age influence water yield. For this reason, we could attribute changes in water yield to changes in water use by the forested ecosystem. These unique aspects of our study design permitted us to draw inferences about resilience of headwater forested catchments to climate warming and environmental and ecological factors that may influence this response.

### Factors that influence elasticity

Both hydrological and ecological mechanisms may potentially contribute to forest expressions of elasticity in response to climate warming (i.e., an increase in the DI). Hydrological mechanisms involve changes in the accessibility of water storages for ET, whereas ecological mechanisms involve changes in forest composition, structure, and function that affect water use. Future research should focus on which mechanisms are likely to dominate under different conditions.

Hydrological factors influencing elasticity include *P* and ET. Total annual changes in precipitation were variable among the catchments (with some showing an increase, a decrease or no change); however, partitioning catchments according to the degree of change in precipitation did not have an effect on the relationship between dynamic deviation and either degree of warming or elasticity. In contrast, the timing or seasonality of *P* and ET within a year did have an effect. Gentine *et al*. (2012) and Williams *et al*. (2012) used a Budyko framework to show that strongly seasonal precipitation contributed to higher evaporative indices. Based on the geographic distribution of headwater catchments in this study, our findings suggest that the seasonality of *P* and ET may also explain elasticity in water-yield responses to climate, with smaller responses of EI to DI in catchments where precipitation has less seasonality. For example, the eastern catchments (CWT, DOR, ELA, FER, HBR, KEJ, MAR, TLW) generally had summer *P*, synchronized *P* and ET (Yokoo *et al.*, 2008), transpiration limited more by atmospheric evaporative demand than by soil water availability, and/or shallow slopes with deeper soils where water residence times are relatively long (Voepel *et al.*, 2011). These eastern catchments tended to have small changes in water yields relative to variation in energy inputs (especially CWT, ELA, HBR, MAR). A potential change in ET could have been masked by deep soils and high baseflow, but there did not seem to be a consistent pattern in properties among the eastern catchments (e.g., FER has shallow soils, MAR has substantial loss of water to regional groundwater aquifers). The western catchments (AND, CAR, LVW, NWT, UPC) generally had winter-dominated *P*, desynchronized *P* and ET, transpiration limited more by soil water availability than by atmospheric evaporative demand, and/or steep slopes with shallow soils where water residence times are relatively short (McGuire *et al.*, 2005). These western sites tended to have more water-yield change in response to variation in energy inputs (especially CAR, NWT, LVW, UPC).

Another hydrological factor influencing elasticity was altered access to physical storages of water (in ice, groundwater, etc.). The alpine sites (e.g., NWT and LVW) had among the lowest elasticity values and the most negative dynamic deviation values, indicating that these ecosystems had low resilience. Water yield at these sites likely responded strongly to climate warming through increased melting of the water stored in glaciers, permafrost, and seasonal snowpacks (Baron *et al.*, 2009; Caine, 2011), as suggested by many studies (Barnett *et al.*, 2008; Stewart, 2009; Trujillo *et al.*, 2012).

Ecological factors also influence elasticity and water yield responses to climate warming. Our study catchments varied in their ecological properties, including phenology and the sensitivity of stomatal resistance to soil water availability and atmospheric evaporative demand (e.g., Ewers *et al.*, 2006; Grant *et al.*, 2009). In general, water yield tended to increase with warming at conifer catchments [Fig.8; Table1, but see comment on CWT 17 (ID #3a) below], perhaps because of stomatal control of transpiration or lagged phenologic response to increased soil moisture from snow/ice melt (Grier & Running, 1977; Chabot & Hicks, 1982). In contrast, water yield tended to decrease with warming at deciduous catchments, perhaps because trees were able to leaf out earlier in response to warming or because of species-specific responses of transpiration to atmospheric evaporative demand (Swank *et al.*, 2001; Ford *et al.*, 2011; Polgar & Primack, 2011). Mixed forests responded to warming in a manner consistent with the combined responses of conifer and deciduous forests.

We recognize the potential importance of forest age (e.g., Cornish & Vertessy, 2001), but we were constrained in our ability to assess the role of forest age in conferring hydrologic resilience because our catchments included few old forests. However, the oldest forest (∼500 years) and younger more diverse forests had larger elasticity (*e *>* *1), whereas the younger and less diverse forests exhibited smaller elasticity (*e *<* *1). Among these younger forests, conifer forests appeared less able to adapt and take advantage of warmer conditions by increasing ET (thereby leading to larger water yields), and deciduous forests appeared more able to adapt (therefore leading to smaller water yields) in these energy-limited sites. Carbon dioxide fertilization effects may also have influenced transpiration (Bolker *et al.*, 1995).

Forest catchments varied in their water-yield (EI) responses to changes in available energy (DI). In the alpine catchments, EI varied a great deal relative to changes in energy inputs (showing low elasticity) because transpiration is limited by dry, short summers. In these catchments, climate warming led to increased water yield because the ecosystems could not adjust over the short term and because stored water melted (we define this as no resilience). The conifer forests included catchments with the widest variation in EI, which varied considerably in response to changes in DI (showing low elasticity) perhaps because transpiration is limited by reduced vapor pressure gradients and/or soil water availability, and therefore is unresponsive to changes in temperature (less resilient). The deciduous forests included catchments where EI varied relatively little despite changes in energy inputs (showing high elasticity). Most of these forests experience wet summers, so transpiration is not limited by water, and leaf area, timing of leaf out and leaf fall can respond to interannual variation in temperature (more resilient). Counter to the general trend, the coniferous catchment at CWT [CWT 17 (ID #3a)] had greater elasticity than the deciduous catchment [CWT 18 (ID #3b)], likely because it had been cut and replanted with a conifer plantation 60 years ago and was still relatively young. Young conifer forests are less able to regulate water use than older conifers (Moore *et al.*, 2004; Ford *et al.*, 2011). In mixed forests, EI varied the least in response to changes in energy inputs (highest elasticity and resilience). Diverse forest types and older forest systems appeared to show greater hydrologic resilience, perhaps because older forests have been acclimated by past climate variations in DI and associated biophysical responses.

### Management implications

A significant proportion of the water supply for human consumption originates from forested catchments (e.g., 53% in the US; Brown *et al.*, 2008), and these supplies are likely to be impacted by climate warming (Aber *et al.*, 1995). In addition to climate change effects, forest management activities (i.e., deforestation, reforestation and afforestation) may have significant consequences on the hydrological resilience of water yields (Fischer *et al.*, 2006). The direction of impact has been debated. For example, some argue that additional forest cover will reduce water yield, whereas others suggest it will increase water yield by intensifying the hydrological cycle (Ellison *et al.*, 2012). Greater insight to links between climatic variability and forest water yields may help inform this debate.

We observed a significant nonlinear relationship between elasticity and dynamic deviation of water yield in response to climate warming at the 21 study sites. We found that sites with relatively modest climate warming had low elasticity and large negative dynamic deviations. Water yields from forested headwater catchments responded nonuniformly to climate warming. Elastic catchments (*e *>* *1) that remained close to the theoretical Budyko curve in response to climate warming had predictable water-yield changes. In contrast, inelastic catchments (*e *<* *1) showed substantial deviations from the Budyko curve in response to climate warming and had unpredictable water yield changes.

Our novel application of the Budyko curve suggests a direction for improving forest management strategies in the face of changing climatic conditions. For example, forest managers will likely want to prioritize forested catchments that are hydrologically resilient to climate warming because replicating naturally resilient ecosystems is so difficult. Furthermore, forest managers will likely need to consider forest type and age as factors that influence hydrologic resilience; further analysis is needed to detect and discriminate the influences of forest type and age on catchment water yields.

## Conclusion

This study indicates that the Budyko framework, using meteorological and discharge data from gauged headwater catchments, may help predict changes in water balance partitioning in response to climate warming. Expert knowledge of the individual catchments indicates that both environmental factors (e.g., summer precipitation, summer length, and water residence time) and ecological factors (forest type and age) contributed to the observed variability in water yield responses to climate warming. Further research into these factors with longer datasets that include a broader range of forest types and age, factors that appear to influence elasticity, would help extend the findings of this article to ungauged headwater catchments.

## Supporting Information

Table S1 Temperature (*T*), precipitation (*P*), potential evapotranspiration (PET, estimated using the Hamon method) and discharge (*Q*) from catchment 5-water-year cool periods (period with lowest average temperature) and 5-water-year warm periods (period with highest average temperature).
