# Supplementary material for: Changing forest water yields in response to climate warming: results from long-term experimental watershed sites across North America
Source: Glob Chang Biol. 2014 Jun 14;20(10):3191–208. doi: 10.1111/gcb.12615 (PMC4282258; doi:10.1111/gcb.12615)
Supplement: Table S1 — Temperature (T), precipitation (P), potential evapotranspiration (PET, estimated using the Hamon method) and discharge (Q) from catchment 5-water-year cool periods (period with lowest average temperature) and 5-water-year warm periods (period with highest average temperature). [file gcb0020-3191-SD1.docx]

Supplementary Table 1. Temperature (T), precipitation (P), potential evapotranspiration (PET, estimated using the Hamon method) and discharge (Q) from catchment 5-water year cool periods (period with lowest average temperature) and 5-water year warm periods (period with highest average temperature).

| **ID** | **Catchment** | **Cool Period** | | | | | **Warm Period** | | | | |
| --- | --- | --- | --- | --- | --- | --- | --- | --- | --- | --- | --- |
|  |  | **Year** | **T (°C)** | **P (mm)** | **PET (mm)** | **Q (mm)** | **Year** | **T (°C)** | **P (mm)** | **PET (mm)** | **Q (mm)** |
| 1a | AND 2 | 1982 | 8.6 | 2757.5 | 581.5 | 1796.2 | 1988 | 9.4 | 1960.4 | 629.0 | 937.4 |
|  |  | 1983 | 9.2 | 2602.5 | 602.4 | 1443.3 | 1989 | 8.8 | 2270.4 | 586.4 | 1362.9 |
|  |  | 1984 | 8.9 | 2696.0 | 592.1 | 1586.0 | 1990 | 9.5 | 1964.0 | 621.6 | 994.6 |
|  |  | 1985 | 8.6 | 2079.7 | 594.3 | 1143.1 | 1991 | 8.9 | 1928.1 | 593.2 | 971.0 |
|  |  | 1986 | 9.0 | 2378.6 | 600.4 | 1275.0 | 1992 | 10.6 | 1710.7 | 668.9 | 770.7 |
| 1b | AND 8 | 1982 | 8.6 | 2757.5 | 581.5 | 1633.1 | 1988 | 9.4 | 1960.4 | 629.0 | 800.9 |
|  |  | 1983 | 9.2 | 2602.5 | 602.4 | 1481.5 | 1989 | 8.8 | 2270.4 | 586.4 | 1111.7 |
|  |  | 1984 | 8.9 | 2696.0 | 592.1 | 1485.7 | 1990 | 9.5 | 1964.0 | 621.6 | 886.5 |
|  |  | 1985 | 8.6 | 2079.7 | 594.3 | 1111.2 | 1991 | 8.9 | 1928.1 | 593.2 | 876.6 |
|  |  | 1986 | 9.0 | 2378.6 | 600.4 | 1147.4 | 1992 | 10.6 | 1710.7 | 668.9 | 626.9 |
| 2 | CAR | 1985 | 9.3 | 2278.8 | 598.7 | 1462.5 | 1991 | 10.2 | 3382.1 | 619.9 | 2505.0 |
|  |  | 1986 | 9.8 | 2867.7 | 615.9 | 1921.8 | 1992 | 11.2 | 2682.6 | 665.2 | 2534.9 |
|  |  | 1987 | 10.7 | 3029.5 | 638.3 | 2018.8 | 1993 | 11.3 | 2665.3 | 667.5 | 2026.2 |
|  |  | 1988 | 9.3 | 2587.5 | 580.9 | 1601.8 | 1994 | 10.9 | 2776.4 | 638.2 | 2829.4 |
|  |  | 1989 | 9.0 | 2484.4 | 591.5 | 1637.6 | 1995 | 11.6 | 3260.8 | 677.5 | 2639.7 |
| 3a | CWT 17 | 1977 | 11.9 | 1837.0 | 737.1 | 711.0 | 1989 | 13.2 | 2187.8 | 766.9 | 667.0 |
|  |  | 1978 | 11.9 | 1581.2 | 727.0 | 683.6 | 1990 | 13.4 | 2015.5 | 787.4 | 954.0 |
|  |  | 1979 | 12.4 | 2233.7 | 739.3 | 974.7 | 1991 | 14.1 | 1958.7 | 816.4 | 699.6 |
|  |  | 1980 | 12.8 | 1934.7 | 769.9 | 974.8 | 1992 | 12.9 | 1891.6 | 752.5 | 613.7 |
|  |  | 1981 | 12.1 | 1238.6 | 742.6 | 186.9 | 1993 | 13.1 | 1937.8 | 784.5 | 971.1 |
| 3b | CWT 18 | 1977 | 11.9 | 1837.0 | 737.1 | 1046.6 | 1989 | 13.2 | 2187.8 | 766.9 | 1068.4 |
|  |  | 1978 | 11.9 | 1581.2 | 727.0 | 1039.2 | 1990 | 13.4 | 2015.5 | 787.4 | 1492.2 |
|  |  | 1979 | 12.4 | 2233.7 | 739.3 | 1355.2 | 1991 | 14.1 | 1958.7 | 816.4 | 1186.3 |
|  |  | 1980 | 12.8 | 1934.7 | 769.9 | 1473.9 | 1992 | 12.9 | 1891.6 | 752.5 | 1009.3 |
|  |  | 1981 | 12.1 | 1238.6 | 742.6 | 466.4 | 1993 | 13.1 | 1937.8 | 784.5 | 1439.3 |
| 4a | DOR HP3 | 1992 | 3.6 | 997.6 | 421.8 | 559.6 | 1998 | 6.1 | 795.0 | 485.2 | 336.6 |
|  |  | 1993 | 3.9 | 962.4 | 448.6 | 619.5 | 1999 | 5.7 | 817.7 | 507.9 | 339.0 |
|  |  | 1994 | 3.3 | 943.1 | 452.8 | 532.8 | 2000 | 4.9 | 1026.4 | 484.0 | 581.6 |
|  |  | 1995 | 5.3 | 1112.1 | 491.1 | 661.6 | 2001 | 4.9 | 936.8 | 482.1 | 398.0 |
|  |  | 1996 | 3.6 | 1241.1 | 457.1 | 731.6 | 2002 | 6.4 | 1074.4 | 511.1 | 578.6 |
| 4b | DOR HP3A | 1992 | 3.6 | 997.6 | 421.8 | 541.5 | 1998 | 6.1 | 795.0 | 485.2 | 342.7 |
|  |  | 1993 | 3.9 | 962.4 | 448.6 | 583.9 | 1999 | 5.7 | 817.7 | 507.9 | 311.3 |
|  |  | 1994 | 3.3 | 943.1 | 452.8 | 502.3 | 2000 | 4.9 | 1026.4 | 484.0 | 681.5 |
|  |  | 1995 | 5.3 | 1112.1 | 491.1 | 642.7 | 2001 | 4.9 | 936.8 | 482.1 | 394.1 |
|  |  | 1996 | 3.6 | 1241.1 | 457.1 | 755.6 | 2002 | 6.4 | 1074.4 | 511.1 | 655.2 |
| 4c | DOR HP4 | 1992 | 3.6 | 997.6 | 421.8 | 527.0 | 1998 | 6.1 | 795.0 | 485.2 | 293.8 |
|  |  | 1993 | 3.9 | 962.4 | 448.6 | 600.1 | 1999 | 5.7 | 817.7 | 507.9 | 286.5 |
|  |  | 1994 | 3.3 | 943.1 | 452.8 | 526.3 | 2000 | 4.9 | 1026.4 | 484.0 | 551.7 |
|  |  | 1995 | 5.3 | 1112.1 | 491.1 | 635.7 | 2001 | 4.9 | 936.8 | 482.1 | 309.3 |
|  |  | 1996 | 3.6 | 1241.1 | 457.1 | 721.1 | 2002 | 6.4 | 1074.4 | 511.1 | 704.3 |
| 4d | DOR HP5 | 1992 | 3.6 | 997.6 | 421.8 | 564.5 | 1998 | 6.1 | 795.0 | 485.2 | 336.5 |
|  |  | 1993 | 3.9 | 962.4 | 448.6 | 664.7 | 1999 | 5.7 | 817.7 | 507.9 | 402.8 |
|  |  | 1994 | 3.3 | 943.1 | 452.8 | 525.6 | 2000 | 4.9 | 1026.4 | 484.0 | 474.5 |
|  |  | 1995 | 5.3 | 1112.1 | 491.1 | 694.9 | 2001 | 4.9 | 936.8 | 482.1 | 322.7 |
|  |  | 1996 | 3.6 | 1241.1 | 457.1 | 824.1 | 2002 | 6.4 | 1074.4 | 511.1 | 631.2 |
| 4e | DOR PC | 1992 | 4.1 | 1024.6 | 461.1 | 536.7 | 1998 | 6.7 | 793.9 | 549.0 | 360.5 |
|  |  | 1993 | 4.4 | 994.9 | 489.6 | 551.6 | 1999 | 6.6 | 926.8 | 562.3 | 338.5 |
|  |  | 1994 | 3.5 | 877.5 | 483.4 | 436.8 | 2000 | 5.4 | 1078.4 | 506.6 | 645.3 |
|  |  | 1995 | 5.6 | 894.9 | 534.4 | 485.0 | 2001 | 5.4 | 889.1 | 529.1 | 296.7 |
|  |  | 1996 | 4.1 | 1268.4 | 487.9 | 759.1 | 2002 | 6.8 | 952.2 | 555.3 | 624.7 |
| 5 | ELA | 1993 | 2.1 | 645.3 | 447.0 | 182.8 | 1998 | 5.3 | 657.5 | 526.2 | 140.1 |
|  |  | 1994 | 1.7 | 681.6 | 473.7 | 137.1 | 1999 | 3.9 | 848.9 | 494.9 | 277.0 |
|  |  | 1995 | 3.8 | 612.7 | 506.8 | 175.2 | 2000 | 3.8 | 843.2 | 482.8 | 336.7 |
|  |  | 1996 | 1.3 | 916.7 | 451.8 | 297.6 | 2001 | 3.2 | 1005.0 | 510.2 | 406.3 |
|  |  | 1997 | 1.8 | 725.3 | 482.8 | 297.1 | 2002 | 3.7 | 738.0 | 510.3 | 262.4 |
| 6 | FER | 1977 | 8.1 | 1371.8 | 561.4 | 572.8 | 1987 | 10.0 | 1262.1 | 628.8 | 500.9 |
|  |  | 1978 | 8.1 | 1428.8 | 581.2 | 715.0 | 1988 | 9.9 | 1256.9 | 655.8 | 479.3 |
|  |  | 1979 | 8.5 | 1554.2 | 578.3 | 738.4 | 1989 | 9.3 | 1645.7 | 607.3 | 812.7 |
|  |  | 1980 | 9.3 | 1511.8 | 621.9 | 759.6 | 1990 | 9.5 | 1484.4 | 636.0 | 669.8 |
|  |  | 1981 | 8.6 | 1515.1 | 616.6 | 694.3 | 1991 | 11.1 | 1314.3 | 688.9 | 600.3 |
| 7a | HBR 3 | 1992 | 4.9 | 1344.4 | 451.8 | 887.1 | 1998 | 6.8 | 1392.1 | 491.7 | 901.9 |
|  |  | 1993 | 5.4 | 1248.8 | 485.1 | 782.9 | 1999 | 6.9 | 1422.7 | 529.5 | 866.0 |
|  |  | 1994 | 4.6 | 1338.7 | 486.6 | 853.4 | 2000 | 6.0 | 1326.7 | 502.4 | 934.4 |
|  |  | 1995 | 6.2 | 1077.7 | 495.6 | 597.0 | 2001 | 6.2 | 1104.1 | 526.4 | 626.9 |
|  |  | 1996 | 5.3 | 1722.7 | 475.5 | 1384.9 | 2002 | 7.2 | 1204.7 | 514.7 | 705.0 |
| 7b | HBR 6 | 1992 | 3.7 | 1380.9 | 411.4 | 892.8 | 1998 | 5.5 | 1465.5 | 457.6 | 987.2 |
|  |  | 1993 | 3.9 | 1324.4 | 435.0 | 833.6 | 1999 | 5.8 | 1555.3 | 477.6 | 989.7 |
|  |  | 1994 | 3.3 | 1404.6 | 422.1 | 900.8 | 2000 | 4.7 | 1409.0 | 448.9 | 1003.7 |
|  |  | 1995 | 4.2 | 1142.6 | 423.7 | 651.8 | 2001 | 5.0 | 1155.4 | 494.6 | 703.3 |
|  |  | 1996 | 4.3 | 1862.2 | 457.1 | 1518.3 | 2002 | 6.2 | 1266.8 | 490.1 | 759.4 |
| 8 | LVW | 1995 | 0.8 | 1285.7 | 186.3 | 992.5 | 2000 | 2.8 | 858.6 | 268.1 | 625.9 |
|  |  | 1996 | 1.3 | 1175.5 | 227.0 | 995.3 | 2001 | 1.6 | 851.4 | 238.6 | 759.8 |
|  |  | 1997 | 0.9 | 1295.1 | 222.3 | 976.0 | 2002 | 2.0 | 595.6 | 243.7 | 517.9 |
|  |  | 1998 | 0.9 | 905.3 | 219.5 | 432.0 | 2003 | 1.5 | 897.7 | 231.5 | 1064.0 |
|  |  | 1999 | 1.0 | 996.7 | 180.4 | 908.2 | 2004 | 1.4 | 924.9 | 235.3 | 397.7 |
| 9a | MAR 2 | 1993 | 2.6 | 758.2 | 443.7 | 164.2 | 1998 | 6.0 | 690.5 | 518.5 | 88.0 |
|  |  | 1994 | 2.5 | 841.3 | 476.1 | 185.3 | 1999 | 5.0 | 1073.3 | 508.0 | 303.1 |
|  |  | 1995 | 4.2 | 819.5 | 499.1 | 135.8 | 2000 | 4.9 | 569.7 | 518.2 | 71.9 |
|  |  | 1996 | 1.7 | 792.0 | 470.7 | 223.6 | 2001 | 3.7 | 848.9 | 512.5 | 270.9 |
|  |  | 1997 | 2.8 | 908.7 | 479.1 | 208.2 | 2002 | 5.0 | 851.6 | 521.5 | 246.5 |
| 9b | MAR 5 | 1993 | 1.8 | 785.4 | 416.3 | 114.1 | 1998 | 6.0 | 682.4 | 517.6 | 47.7 |
|  |  | 1994 | 1.4 | 814.7 | 439.3 | 120.4 | 1999 | 5.0 | 1140.3 | 506.0 | 233.3 |
|  |  | 1995 | 3.3 | 823.0 | 470.2 | 80.0 | 2000 | 5.1 | 611.9 | 527.0 | 50.6 |
|  |  | 1996 | 1.3 | 778.8 | 473.0 | 177.0 | 2001 | 3.9 | 847.4 | 517.6 | 191.2 |
|  |  | 1997 | 2.8 | 910.6 | 482.3 | 146.5 | 2002 | 5.2 | 745.3 | 535.3 | 100.2 |
| 10 | NWT | 1992 | -3.2 | 1210.1 | 198.7 | 823.6 | 2000 | -1.8 | 1436.6 | 239.1 | 845.5 |
|  |  | 1993 | -3.7 | 1342.8 | 192.1 | 905.2 | 2001 | -3.1 | 1141.7 | 226.0 | 910.0 |
|  |  | 1994 | -2.9 | 1402.6 | 242.0 | 810.0 | 2002 | -2.8 | 982.0 | 227.9 | 694.8 |
|  |  | 1995 | -3.9 | 1639.0 | 162.0 | 1003.8 | 2003 | -3.7 | 1165.0 | 198.6 | 1076.1 |
|  |  | 1996 | -3.6 | 1407.4 | 211.0 | 963.9 | 2004 | -2.6 | 1067.0 | 190.8 | 849.8 |
| 11a | TLW 35 | 1992 | 3.6 | 1413.6 | 423.4 | 812.1 | 1998 | 6.6 | 962.6 | 518.5 | 359.9 |
|  |  | 1993 | 3.9 | 1438.6 | 454.0 | 826.9 | 1999 | 6.2 | 1281.5 | 534.7 | 494.9 |
|  |  | 1994 | 3.1 | 1249.1 | 461.6 | 640.4 | 2000 | 5.5 | 1267.0 | 510.2 | 523.3 |
|  |  | 1995 | 5.7 | 1198.2 | 517.8 | 530.8 | 2001 | 5.1 | 977.6 | 520.9 | 428.5 |
|  |  | 1996 | 3.2 | 1480.4 | 461.6 | 744.0 | 2002 | 5.8 | 1460.1 | 513.0 | 733.6 |
| 11b | TLW 38 | 1992 | 3.6 | 1413.6 | 423.4 | 779.6 | 1998 | 6.6 | 962.6 | 518.5 | 357.2 |
|  |  | 1993 | 3.9 | 1438.6 | 454.0 | 796.4 | 1999 | 6.2 | 1281.5 | 534.7 | 606.1 |
|  |  | 1994 | 3.1 | 1249.1 | 461.6 | 644.5 | 2000 | 5.5 | 1267.0 | 510.2 | 565.1 |
|  |  | 1995 | 5.7 | 1198.2 | 517.8 | 450.4 | 2001 | 5.1 | 977.6 | 520.9 | 429.0 |
|  |  | 1996 | 3.2 | 1480.4 | 461.6 | 693.9 | 2002 | 5.8 | 1460.1 | 513.0 | 765.9 |
| 12 | UPC | 1995 | -1.2 | 676.3 | 224.8 | 344.3 | 2002 | -1.3 | 569.0 | 225.2 | 475.6 |
|  |  | 1996 | -1.9 | 724.1 | 188.3 | 456.7 | 2003 | 0.0 | 489.7 | 242.4 | 244.2 |
|  |  | 1997 | -1.5 | 866.0 | 231.4 | 570.3 | 2004 | -0.4 | 838.2 | 269.0 | 369.4 |
|  |  | 1998 | 0.4 | 690.7 | 257.5 | 447.3 | 2005 | -0.6 | 641.9 | 225.1 | 419.6 |
|  |  | 1999 | -1.2 | 803.3 | 208.5 | 459.5 | 2006 | -0.6 | 770.8 | 259.7 | 428.0 |
